# Heterogeneous Impacts of Basic Social Health Insurance on Medical Expenditure: Evidence from China’s New Cooperative Medical Scheme

**DOI:** 10.3390/healthcare7040131

**Published:** 2019-11-03

**Authors:** Conglong Fang, Chaofei He, Scott Rozelle, Qinghua Shi, Jiayin Sun, Ning Yu

**Affiliations:** 1 Antai College of Economics and Management, Shanghai Jiao Tong University, Shanghai 200030, China; fcl588@sjtu.edu.cn (C.F.); shq@sjtu.edu.cn (Q.S.); 2 School of Finance, Nanjing Audit University, Nanjing 211815, China; 3 Rural Education Action Program, Freeman Spogli Institute for International Studies, Stanford University, Stanford, CA 94305, USA; rozelle@stanford.edu (S.R.); ningyu@stanford.edu (N.Y.); 4 Department of Statistics, University of British Columbia, Vancouver, BC V6T1Z4, Canada; jiayin.sun@alumni.ubc.ca; 5 Institute for Social and Economic Research, Nanjing Audit University, Nanjing 211815, China

**Keywords:** New Cooperative Medical Scheme (NCMS), medical expenditure, health insurance, China

## Abstract

This paper examines the effects of China’s New Cooperative Medical Scheme (NCMS) on medical expenditure. Utilizing the quasi-random rollout of the NCMS for a difference-in-difference analysis, we find that the NCMS increased medical expenditure by 12.3%. Most significantly, the good-health group witnessed a 22.1% rise in medical expenditure, and the high-income group saw a rise of 20.6%. The effects, however, were not significant among the poor-health or low-income groups. The findings are suggestive of the need for more help for the very poor and less healthy.

## 1. Introduction

With the rapid growth of China’s economy, in 2003, the Chinese government launched a substantial public health insurance project, the New Cooperative Medical Scheme (NCMS). In 2014, the medical expenses of 800 million enrollees amounted to 289.04 billion yuan—70% of which were subsidized by the Chinese government. 

There is compelling evidence rejecting the null hypothesis that medical utilization is not affected by health insurance [1]. Many studies are evaluating the average effect of the NCMS on medical expenditure [2,3].

However, the low-income or poor-health groups may not go to the hospital even if they have joined the NCMS. The World Health Survey of 2003 from 39 medium- and low-income countries showed that transportation costs accounted for over 10% of the total medical expense [4]. The cost of accommodation and food for caretakers was also a big burden [5]. Furthermore, in the first few years of the NCMS, patients were asked to pay the total medical expense by themselves in advance, and then got reimbursement later. The low-income or poor-health groups might not be able to afford the total medical expense in advance.

Thus, it is imperative to study the influence of health insurance on the medical expenditure of different groups. In particular, we explore the extent to which health insurance helps those most in need: low-income or poor-health groups.

This paper contributes to the literature in the following ways. First, we explore the heterogeneous effects of the NCMS among different health status and income groups. Second, from the perspective of income budget constraint, we explain the reasons why poor and less healthy farmers do not go to the hospital even though they have health insurance. Third, our data are from the longitudinal National Rural Fixed-Point Survey (NFS) which covers more provinces and waves than the other two popular surveys, the China Family Panel Studies (CFPS) and the China Health and Retirement Longitudinal Study (CHARLS). Most importantly, based on the quasi-natural experiment of the NCMS, which was rolled out gradually throughout China, we demonstrate a clean and robust result for the causal relationship between health insurance and medical expenditure.

The remainder of this paper is organized as follows. Section 2 provides a background of the NCMS. Section 3 proposes a hypothesis to explain why low-income or poor-health groups who have joined the NCMS might not go to the hospital even if they have joined the NCMS. Section 4 presents the identification strategy and data sources. Section 5 presents the main results as well as the robustness checks. Section 6 provides the heterogeneous results. Section 7 discusses the limitations of this paper. Finally, Section 8 concludes and offers three suggestions for the policy makers.

## 2. Background: The NCMS

Since 1982, there has been two rounds of rural reform. The first round was from 1982 to 1986. The second round began in 2003. The first-round reform significantly promoted the development of rural economy. It mainly included establishing a household contract responsibility system and abolishing planned purchase and supply by the state of agricultural products.

However, after that period, the government shifted its focus from the rural areas to the urban areas. As a result, grain products decreased continually for five years. Thus, farmers’ income grew slowly, and the income gap between urban and rural areas continued to widen greatly.

Therefore, in 2003, to improve farmers’ income and welfare, the Chinese central government launched a new round of rural reform, including the construction of a new cooperative medical scheme, known as the NCMS.

In July 2003, the Chinese central government required local governments to choose at least two counties to implement the NCMS. From 2004 to 2008, the number of participants increased by approximately tenfold. In 2008, the NCMS covered almost all the registered rural population.

As for the total premium, the Chinese governments’ subsidy accounted for the most. The ratio increased from 50% in 2004 to 80% in 2008. In general, the total premium was 20 yuan per person in 2004, and 100 yuan in 2008. Then, from 2008 to 2013, the total premium increased approximately fourfold. The premium paid by each enrollee was 10 yuan in 2004, and 20 yuan in 2008. That is to say, the central and local governments subsidized every enrollee 10 yuan in 2004, and 80 yuan in 2008.

The reimbursement of the NCMS is shown in Table 1. Each person’s reimbursement accounted for over 70% of the total premium. During the period 2004–2007, the lowest ratio of each person’s reimbursement to the total premium was 72.94% in 2006, and the highest was 81.95% in 2005. The total expenditure for the NCMS—of which the governments’ subsidies accounted for the most—reached 346.63 billion yuan in 2007.

However, the person-time of reimbursed beneficiaries was not higher than the number of participants. The former accounted for 95.00% of the latter in 2004, and the ratio decreased to 62.40% until 2007.

At first, this program reimbursed policy holders for mainly inpatient expenses but not at a high level. In 2006, the reimbursement rate was 49% of inpatient expenses, with a ceiling of 14,838 yuan, and outpatient expenses were not covered [6]. Further, the reimbursement rate decreased when enrollees went to high-level hospitals, especially provincial hospitals.

## 3. Hypothesis

On the basis of the theoretical model proposed by Zhao, Zang and Yin [7], we investigated the impact of income budget constraint on farmers’ choosing medical treatment behaviors. Suppose the utility of choosing going to the hospital is u(z−m−e+b) when a farmer who has joined the NCMS is sick and the utility of not going to the hospital is $\bar{u}$.

z is the income when the farmer is sick, m represents the out-of-pocket medical expenses of going to the hospital, b is the net reimbursement of going to the hospital (the premium of the NCMS deducted already), and e is the transportation and accommodation expenses for going to the hospital for farmers.

Theoretically, when $u(z-m-e+b)>\bar{u}$, farmers will go to the hospital when they are sick. However, whether farmers go to the hospital or not is also subject to the income budget constraints.

When z < m + e, which means farmer’s income is lower than the sum of out-of-pocket medical expenses, relative transportation and accommodation expenses, farmers will not go to the hospital. Due to the insufficient medical service capability of local township health centers, when farmers get seriously ill, they have to choose county-level hospitals or above for medical treatment. Therefore, in addition to medical expenses, certain transportation and accommodation expenses are needed.

When m+e<z<m+e+b, which means farmer’s income is higher than the out-of-pocket medical expenses and relative transportation and accommodation expenses, but lower than that when pre-paid costs are considered, farmers will not go to the hospital. In the early implementation of the NCMS, farmers need to pay all medical expenses in advance, and then obtain reimbursement after they go back. Many low-income or less healthy farmers cannot afford this. In this case, z>m+e+b, and the farmer can afford the total medical expense—only then will they go to the hospital.

Some may consider that if farmers can borrow money, they seem to be able to afford pre-paid costs. However, in reality, low-income or less healthy farmers are generally less able to borrow because of the high risk of default. Using a random selection of 3000 rural households throughout China in 2003, Zhu and Li [8] found that only 4.4% of rural households borrowed money from the formal financial institutions, and 18.6% of rural households borrowed money from the informal private sector. They pointed out that information asymmetry and government intervention were the main barriers of credit constraint for farmers. Using the data from the China Health and Retirement Longitudinal Study (CHARLS) of 2013, Chen and Zeng [9] found that poor farmers borrowed less, no matter formal or informal borrowing, mainly because they were less able to earn money to repay. Using the same data, Chen and Zeng [10] found that the per capita formal borrowing of disabled farmers was 38,225.38 yuan, and that of nondisabled farmers was 60,027.60 yuan. The per capita informal borrowing of disabled farmers was 31,039.63 yuan, and that of nondisabled farmers was 36,337.49 yuan. Although the formal borrowing of disabled farmers was greater than the informal borrowing, the incidence of formal borrowing was 7.14%, and that of informal borrowing was 18.77%. This means that only a few disabled farmers could borrow money through formal financial institutions. Therefore, it is hard for the low-income or poor-health farmers to pay the pre-paid costs through borrowing money from others.

To sum up, although farmers have joined the NCMS, those with low income or poor health are still faced with income budget constraints, including out-of-pocket medical expenses, transportation and accommodation expenses, and pre-paid costs, as stated above. All of these may decrease farmers’ intention of going to the hospital.

## 4. Methodology and Data

### 4.1. Specification

Following the literature [11], we use a difference-in-difference (DID) method to identify the intention to treat (ITT) effect of the NCMS on medical expenditure. Our estimated equation is as follows:

(1) $$Medical_{ict}=\beta_{0}+\beta_{1}NCMS_{ct}+\beta_{2}X_{ict}+\theta_{i}+\mu_{t}+\varepsilon_{ict}$$

The dependent variable $Medical_{ict}$ is the medical expenditure (in log) of a household i in village c. $NCMS_{ct}$ is an indicator of whether a village implemented the NCMS. $X_{ict}$ is a vector of time-varying variables, including health status, age of the head of household, household level per capita income, county-level population, county-level Gross Domestic Product (GDP) and the interaction of dummy variables of poor village and year. $\theta_{i}$ and $\mu_{t}$ are vectors of household and year dummy variables.

### 4.2. Data and Variables

The data used in this paper come from the longitudinal National Rural Fixed-Point Survey (NFS) conducted by the Ministry of Agriculture of China. The NFS covers approximately 300 villages and 31 provinces every year. One representative village was randomly chosen in each county after a multi-stage random sampling process. Between 40 and 120 households were randomly chosen in each village [12]. The NFS includes household-level and village-level questionnaires.

The NFS is one of the biggest and most authoritative surveys of rural residents in China. The National Bureau of Statistics also conducts a survey of rural residents, which is more representative and has a large sample size. However, its sample changed every three years and is not open to researchers. Overall, the microdata in China is relatively hard to achieve. Therefore, since 2010, some universities began to conduct surveys by themselves, such as the China Family Panel Studies (CFPS), China Health and Retirement Longitudinal Survey (CHARLS) and the China Household Finance Survey (CHFS).

The construction of the $NCMS_{ct}$. Using the village-level questionnaire, we view a village as a piloted village if at least one household in the village joined the NCMS. The significant impact of the NCMS on medical expenditure still exists when we use different cut-offs to construct the dummy variable of the NCMS in robustness checks. Since the NCMS was announced in July 2003 but began to reimburse from 2004, we view year 2003 as the base year when there were no villages that had implemented the NCMS, following Bai and Wu [6].

We use the years 2003 to 2007 to generate results. There are three reasons that the period from 2003 to 2007 is appropriate. First, from 2003, the NFS has added more variables, including individual age and health status. Second, since the NCMS was implemented gradually throughout China between 2003 and 2007, using the difference-in-difference method could estimate a clean and accurate result. After 2007, almost all households were exposed to the NCMS. Therefore, there are no observations in the control group. As a result, the difference-in-difference method could not estimate the difference between the experimental group and the control group after 2007. Third, after 2007, the NCMS did not favor vulnerable groups. This improves the applicability of our conclusions to the present situation. Therefore, the years 2003 to 2007 provide an appropriate period to generate results. Table 2 presents the description, means, and standard deviations of the variables included in our specification.

**Figure 1:**
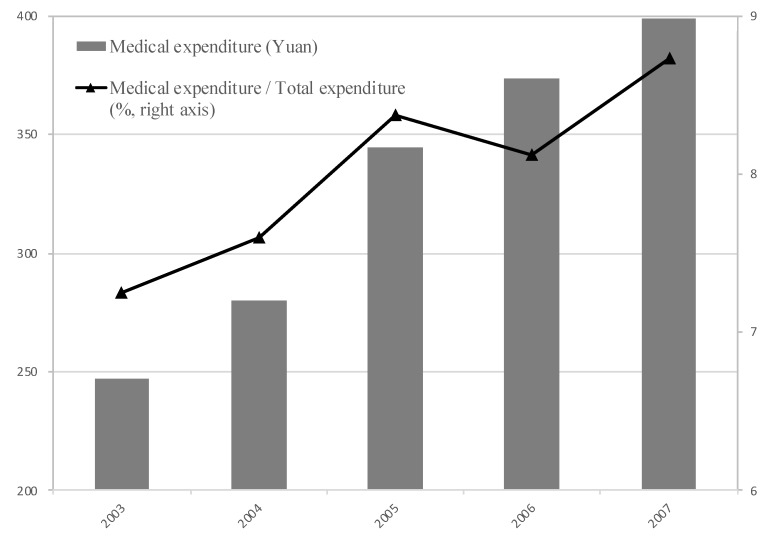
Medical Expenditure from 2003 to 2007.

The medical expenditure increased from 247.12 yuan in 2003 to 398.76 yuan in 2007, by 61.36% (Figure 1). The ratio of the medical expenditure to the total expenditure increased from 7.25% in 2003 to 8.73% in 2007. The increase in medical expenditure might be caused by two factors. First, the implementation of the NCMS in 2003 stimulated the growth of medical expenditure. Second, the growth of income since 2003 has been the foundation of the increase in medical expenditure.

**Figure 2:**
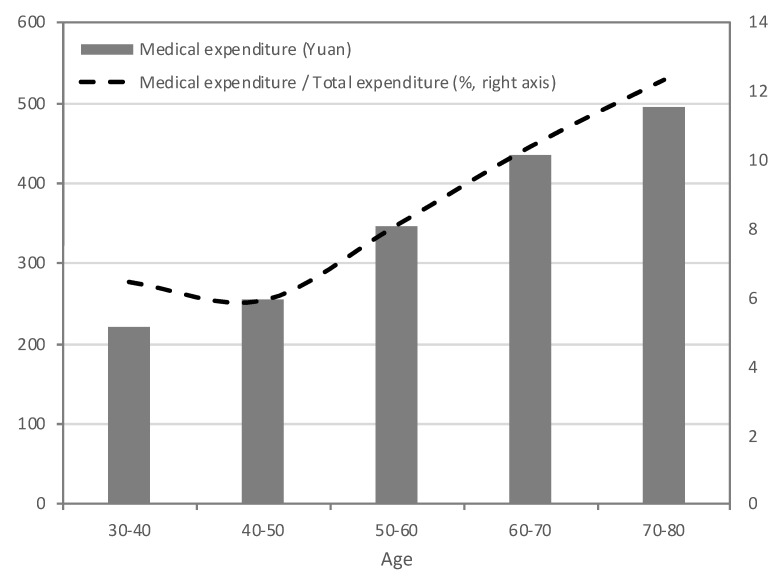
Medical Expenditure over the Lifecycle (2003 to 2007).

The trends of medical expenditure and its share of the total expenditure over the lifecycle are shown in Figure 2. They fit well with the demand for medical service for different age groups. The medical expenditure increased from 227.16 yuan at age 30–40 to 449.87 yuan at age 70–80, by 98.04%. The ratio of medical expenditure to the total expenditure increased from 6.13% at age 30–40 to 11.35% at age 70–80. Predictably, as China is aging, the demand for medical service will increase very fast.

### 4.3. Identification Assumptions

Based on wave 2007 and year 2003, Table 3 examines the assumption of parallel trends. It shows that there are no obvious trends across different waves before the introduction of the NCMS. In addition, there are no significant determinants of county NCMS participation [11,13]. We will perform some robust checks in the next section.

## 5. Basic Results

Column 1 of Table 4 shows that the NCMS increased medical expenditure by 9.5%. In column 2, health status and age are added as additional control variables. As a result, the coefficient decreases slightly. Column 3 shows that the result is consistent after adding some control variables that might affect the selection of the introduction of the NCMS.

The total effect of the NCMS on medical expenditure depends on elasticity. With the NCMS, the cost of medical services decreases, and enrollees consume more medical services. With the increase in medical demand, the price, in turn, should increase. Zhao, Zang and Yin [7], using the data of 122 countries from 2009 and 2010, found that the medical expenditure elasticity of the coinsurance rate was 0.45. Our basic results are consistent with their findings.

Table 5 presents the results using various specifications of Equation 1 as robustness checks. In Table 4, when we construct the variable of the NCMS, we view a village as a piloted village if at least one household in the village joined the NCMS. In column 1 of Table 5, we view a village as a piloted village if at least 5% of the households in the village joined the NCMS. Similarly, in column 2 of Table 5, we view a village as a piloted village if at least 10% of the households in the village joined the NCMS. The results of the first two columns show that different cut-offs do not make a big difference.

In column 3, we trim the variable $Medical_{ict}$ at the 1st and 99th percentiles. The result is still significantly positive at 11.3%, which is close to our estimation in Table 4.

The other three columns control additional control variables. In column 4, we add the county-level fiscal revenue as an additional control variable. In column 5, we add the county-level per capita income of farmers as an additional control variable. In column 6, we add the village -level per capita income of farmers as an additional control variable. Encouragingly, we find that the significant impact of the NCMS on medical expenditure still exists.

## 6. Heterogeneous Results

### 6.1. Different Health Status

Column 1 of Table 6 shows that the NCMS insignificantly increased the medical expenditure among the poor-health group. In contrast, as seen in column 2, the medical expenditure of the good-health group significantly increased by 22.1%, approximately 39.23 yuan.

The National Health Services Survey (NHSS) shows that the hospitalization rate of rural residents doubled from 3.4% in 2003 to 6.8% in 2008. This sharp growth occurred not only in the poor-health group but also in the good-health group. Wagstaff, Lindelow, Gao, Xu and Qian [2] found that insured patients tended to receive more expensive drugs and tests, which results from moral hazard in the Chinese context. Specifically, Babiarz, Miller, Yi, Zhang and Rozelle [14] found that patients’ out-of-pocket spending decreased in the township health center but increased in the county hospital.

### 6.2. Different Income Quantiles

Columns 3 to 5 of Table 6 suggest that the NCMS increased the medical expenditure of the high-income group by 20.6%, but was not significant in the low-income group, the primary target of the NCMS. It could be that poor farmers still cannot afford for the total medical expenditure. The average income of the low-income group was 1507.73 yuan in 2003, which was under the poverty line. Their medical expenditure accounted for 13.0% of their total expenditure, compared with 5.8% for the high-income group.

In addition, the NHSS shows that the hospitalization rate of the high-income group was 15.3% higher than that of the low-income group in 2008. Although 34.6% of low-income residents should have been hospitalized, 81.5% were not due to financial difficulties. The reimbursement rate for hospitalization expenses was 26.9%, approximately 934 yuan. After that, enrollees still had to pay approximately 2537 yuan by themselves, which exceeds their per capita household income of 2466 yuan.

Wagstaff, Lindelow, Gao, Xu and Qian [2] also found that the NCMS increased the utilization of outpatient services at village and township levels among the poorest 20% of their sample. The NCMS, however, increased the utilization of inpatient services among the rest of the sample. The NCMS reimbursed for mainly inpatient services instead of outpatient services. Moreover, the average deductible on medical expenditure at the township clinics in 2006 was 125 yuan [6]. Therefore, it is not surprising that the effect of the NCMS was not significant among the low-income group.

## 7. Discussion

Using data from a large nationally representative longitudinal survey, the results of the difference-in-difference estimation show that China’s NCMS significantly boosted the medical expenditure of rural residents with good health or high income, but not those with poor health or low income who are subject to income budget constraints.

Although the results above are robust with different specifications and consistent with the findings of other researches, there are several limitations which could be improved in the future. First, due to the limitations of data and method, we only used the data from 2003 to 2007, which might be too old to apply to the current situation. We could design an up-to-date survey to estimate the effect of the reimbursement rate of the NCMS on medical expenditure after 2007. Second, the longitudinal National Rural Fixed-Point Survey (NFS) only chooses one village in each county and covers approximately 300 villages every year. Therefore, we could use surveys covering more villages in the future. Third, except for the relative transportation, accommodation expenses, and pre-paid costs, other reasons for the insignificant effect of NCMS on medical expenditure for low-income or poor-health still need to be explored. We could perform some detailed surveys to explore other reasons in the future.

## 8. Conclusions

The empirical results above show that there is no significant evidence supporting the idea that the NCMS simulates the medical expenditure of low-income or poor-health farmers. However, the medical expenditure of good-health farmers increased by 22.1%, and that of high-income farmers increased by 20.6%. The main reason is that poor-health or low-income farmers still could not afford the relative transportation, accommodation expenses, and pre-paid costs. Since the low-income or poor-health farmers are the most in need, the findings are important for the policy makers to improve public health insurance.

As a form of social security, the health insurance program should consider adopting discriminatory policies for the benefit of people with poor health and/or low income, as, for these groups, medical expenditure constitutes a greater financial burden.

The local township health centers or county-level hospitals have higher reimbursement rates than the municipal and provincial hospitals. Therefore, the government should firstly provide more sufficient medical service capability, such as professional doctors and advanced medical equipment, for local township health centers or county-level hospitals, which are more convenient for famers. Second, the government could simplify the reimbursement process as much as possible to reduce the income budget constraints of patients with poor health and/or low income. Third, the government could encourage more benefaction towards patients with poor health and/or low income who need support for long-term care.

## Figures and Tables

**Table 1:** The Reimbursement of the New Cooperative Medical Scheme (NCMS).

Year	Number of Enrollees (million)	Person-time of Reimbursed Beneficiaries (million)	Per Capita Total Premium (yuan)	Per Capita Reimbursement (yuan)	Total Fund Expenditure (billion yuan)
2004	80	76		32.96	26.37
2005	179	122	42.10	34.50	61.75
2006	410	272	52.10	38.00	155.81
2007	726	453	58.90	47.75	346.63

Note. Data are from the China Health Statistics Yearbook. Per Capita Reimbursement = Total Fund Expenditure/Number of Enrollees.

**Table 2:** Description of the Variables (2003 to 2007).

Variable	Description	Mean	SD
Medical	Per capita total medical expenditure (yuan)	321.15	1198.92
NCMS	1 = piloted; 0 = otherwise	0.46	0.50
Income	household level per capita income (yuan)	5403.61	7833.36
Health	Per capita health status: 1 = excellent, 2 = good, 3 = fair, 4 = bad, and 5 = no working capacity	1.64	0.69
Age	Age of the head of household	50.63	11.54
County-level population	Total population (thousand)	61.04	34.17
County-level GDP	Gross Domestic Product of each county (100 million yuan)	61.31	63.70
Poor	1 = poor village; 0 =otherwise	0.09	0.29

**Table 3:** Pre-Trend Test.

Independent Variables	Dependent Variable: Medical (log)
Wave2003*Year2004	0.055 (0.139)
Wave2004*Year2004	−0.039 (0.160)
Wave2005*Year2004	−0.033 (0.129)
Wave2006*Year2004	0.062 (0.119)
Wave2003*Year2005	−0.206 (0.141)
Wave2004*Year2005	−0.196 (0.158)
Wave2005*Year2005	0.017 (0.158)
Wave2006*Year2005	0.045 (0.146)
Wave2003*Year2006	−0.033 (0.191)
Wave2004*Year2006	0.017 (0.190)
Wave2005*Year2006	0.361 * (0.207)
Wave2006*Year2006	0.317 (0.193)
Wave2003*Year2007	−0.063 (0.179)
Wave2004*Year2007	−0.348 (0.243)
Wave2005*Year2007	0.097 (0.179)
Wave2006*Year2007	0.118 (0.211)
*N*	29,613
*R* ^2^	0.034

Note. Wave2003*Year2004 represents the interaction of the dummy variable of wave 2003 and year 2004, and so on. Standard errors are in parentheses, clustered at the village level. * *p* < 0.1.

**Table 4:** Effects of the NCMS on Medical Expenditure.

Dependent Variable: Medical (log)	(1)	(2)	(3)
NCMS	0.095 *	0.128 **	0.123 *
	(0.051)	(0.060)	(0.065)
Health		0.296 ***	0.301 ***
		(0.041)	(0.045)
Age		0.002	0.002
		(0.002)	(0.002)
Log income			0.158 ***
			(0.028)
Log county-level GDP			0.540 **
			(0.231)
County-level population			−0.002
			(0.010)
Poor*Year Fixed Effects			0.000 **
			(0.000)
*N*	51,497	44,071	30,686
*R* ^2^	0.021	0.027	0.033

Note. All regressions control for household and year fixed effects. Standard errors are in parentheses, clustered at the village level. * *p* < 0.1, ** *p* < 0.05, and *** *p* < 0.01.

**Table 5:** Robustness Checks.

Dependent Variable: Medical (log)	(1)	(2)	(3)
	Cut-off (5%)	Cut-off (10%)	Outliers (1%)
NCMS	0.161 **	0.156 **	0.113 *
	(0.065)	(0.064)	(0.065)
*N*	30,686	30,686	30,419
*R* ^2^	0.034	0.034	0.031
Dependent variable: Medical (log)	(4)	(5)	(6)
	Additional control	Additional control	Additional control
NCMS	0.134 **	0.138 **	0.128 *
	(0.066)	(0.068)	(0.065)
Log county fiscal revenue	0.104		
	(0.147)		
Log county per capita income of farmers		0.876 ***	
		(0.219)	
Log village per capita income of farmers			−0.067
			(0.056)
*N*	30,686	26,773	29,744
*R* ^2^	0.034	0.036	0.034

Note. All regressions control for variables of $X_{ict}$ in estimated Equation 1, household and year fixed effects. Standard errors are in parentheses, clustered at the village level. * *p*< 0.1, ** *p*< 0.05, *** *p*< 0.01.

**Table 6:** Effects of NCMS among Different Groups.

Dependent Variable: Medical (log)	Health		Income		
	(1)	(2)	(3)	(4)	(5)
	Poor	Good	Low	Median	High
Mean (2003)	337.42	177.52	222.38	182.63	354.34
NCMS	0.013	0.221 **	0.088	0.102	0.206 **
	(0.078)	(0.102)	(0.109)	(0.071)	(0.088)
*N*	10,518	11,243	5582	6374	4910
*R^2^*	0.026	0.047	0.053	0.045	0.026

Note. Poor-health (good-health) represents the top (bottom) half of the distribution of health status in 2003. The low-income group represented income in the 0 position to the 20th percentile in 2003; the median-income group, 40th to 60th; the high-income group, 80th to 100th. All regressions control for variables of $X_{ict}$ in estimated Equation 1, household and year fixed effects. Standard errors are in parentheses, clustered at the village level. ** *p* < 0.05.

## References

[1] Finkelstein A., Mahoney N., Notowidigdo M.J. (2018). What does (formal) health insurance do, and for whom?. Annu. Rev. Econ..

[2] Wagstaff A., Lindelow M., Gao J., Xu L., Qian J. (2009). Extending health insurance to the rural population: An impact evaluation of China’s New Cooperative Medical Scheme. J. Health Econ..

[3] Cheng L., Liu H., Zhang Y., Shen K., Zeng Y. (2015). The impact of health insurance on health outcomes and spending of the elderly: Evidence from China’s New Cooperative Medical Scheme. Health Econ..

[4] Saksena P., Xu K., Elovainio R., Perrot J. (2010). Health Services Utilization and Out-of-Pocket Expenditure in Public and Private Facilities in Low-Income Countries.

[5] Saksena P., Reyburn H., Njau B., Chonya S., Mbakilwa H., Mills A. (2010). Patient costs for paediatric hospital admissions in Tanzania: A neglected burden?. Health Policy Plan..

[6] Bai C., Wu B. (2014). Health insurance and consumption: Evidence from China’s New Cooperative Medical Scheme. J. Comp. Econ..

[7] Zhao S., Zang W., Yin Q. (2015). The welfare effect of medical insurance. Econ. Res. J..

[8] Zhu X., Li Z. (2006). Analysis of credit rationing by Chinese formal rural finance. J. Quant. Tech. Econ..

[9] Chen Y., Zeng X. (2018). Who is active in the rural credit market: The poor or the rich?. J. Hunan Univ. (Soc. Sci.).

[10] Chen Y., Zeng X. (2017). An analysis on the credit constraints and factors influencing credit behavior of the rural handicapped. Chin. Rev. Financ. Stud..

[11] Yu N.N., Zhu X. (2018). Affordable care encourages healthy living: Theory and evidence from China’s New Cooperative Medical Scheme. Health Econ..

[12] Benjamin D., Brandt L., Giles J. (2005). The evolution of income inequality in rural china. Econ. Dev. Cult. Chang..

[13] Lei X., Lin W. (2009). The New Cooperative Medical Scheme in rural China: Does more coverage mean more service and better health?. Health Econ..

[14] Babiarz K.S., Miller G., Yi H., Zhang L., Rozelle S. (2012). China’s New Cooperative Medical Scheme improved finances of township health centers but not the number of patients served. Health Affair..

